# Racial disparities in women with ST elevation myocardial infarction: A National Inpatient Sample review of baseline characteristics, co‐morbidities, and outcomes in women with STEMI

**DOI:** 10.1002/clc.24068

**Published:** 2023-07-13

**Authors:** Sriviji Senthil Kumaran, Juan Del Cid Fratti, Anjali Desai, Rimmy Garg, Carlos Requeña‐Armas, Pablo Barzallo, Mena Henien, Mansoor Ahmad, Sudhir Mungee, Ekanka Mukhopadhyay, Tinoy Kizhakekuttu

**Affiliations:** ^1^ Department of Cardiology, OSF Healthcare University of Illinois at Peoria Peoria Illinois USA; ^2^ Department of Cardiology UTHSC College of Medicine Chattanooga Chattanooga Tennessee USA

**Keywords:** National Inpatient Sample, outcomes, race, ST‐elevation myocardial infarction, women

## Abstract

**Background:**

A third of the patients admitted with Acute coronary syndrome (ACS) have ST‐elevation myocardial infarction (STEMI). Previous studies showed that females with STEMI have higher mortality than men.

**Hypothesis:**

There exist significant disparities in outcomes among women of different races presenting with STEMI.

**Methods:**

National inpatient sample (NIS) data was obtained from January 2016 to December 2018 for the hospitalization of female patients with STEMI. We compared outcomes, using an extensive multivariate regression analysis amongst women from different races. Our primary outcome was in‐hospital mortality. Secondary outcomes were revascularization use, procedure complications, and healthcare utilization.

**Results:**

Of 202 223 female patients with STEMI; 11.3% were African American, 7.4% Hispanic, 2.4% Asian, and 4.3% another race. In‐hospital mortality was higher in non‐Caucasian groups. African American (adjusted odds ratio [aOR] 1.2; 95% confidence interval [CI]: 1.07–1.30; *p* < .01) and another race (aOR 1.37; 95% CI: 1.15–1.63; *p* < .01) had higher odds of mortality when compared with white women. African American (aOR 0.69; 95% CI: 0.62–0.72; *p* < .01), Hispanics (aOR 0.81; 95% CI: 0.74–0.88; *p* < .01), and Asian (aOR 0.79; 95% CI: 0.69–0.90; *p* < .01) had lower odds of percutaneous intervention (PCI) when compared with whites. African Americans had fewer odds of Coronary Artery Bypass Graft (CABG) and use of Mechanical Circulatory Support (MCS) during the index admission. Non‐Caucasians had more comorbidities, complications, and healthcare utilization costs.

**Conclusion:**

There are significant racial disparities in clinical outcomes and revascularization in female patients with STEMI. African American women have a higher likelihood of mortality among the different races. Females from minority groups are also less likely to undergo PCI.

## Graphical abstract

We describe a retrospective analysis of all women > 18 years who were admitted with the primary diagnosis of ST‐elevation myocardial infarction (STEMI). Data was obtained from the National Inpatient Sample (NIS). Outcomes were analyzed and compared among women of different races. Our study showed that non‐Caucasian women had higher odds of in‐hospital mortality when compared to Caucasian women. It also showed that Non‐Caucasian women had lower odds of undergoing revascularization with both PCI/CABG.

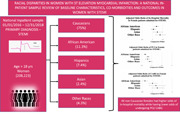

## 1 INTRODUCTION

Cardiovascular disease (CVD) remains the leading cause of death for women in the United States and worldwide despite tremendous advances in the acute management of ST‐elevation myocardial infarction (STEMI) and a significant decline in mortality in the last three decades. Despite the increased prevalence of coronary artery disease (CAD) in Caucasians, research has shown a higher prevalence of cardiovascular risk factors with worse major adverse cardiovascular events (MACE) in the non‐Caucasian population.

Management of acute STEMI has been standardized using evidence‐based guidelines. Emphasis on quality improvement with reimbursement based on performance metrics has ensured adherence to these guidelines and emphasized on early percutaneous intervention (PCI). This has resulted in an improved mortality from 17% in 1985–1990 to 6% in 2000–2008.^1^, ^2^ This change does not span equally among all races and sexes.^3^, ^4^, ^5^, ^6^, ^7^ Prior data have demonstrated that women and minorities are less likely to receive revascularization with higher mortality and increased complications after STEMI.^8^, ^9^ There is not much clarity regarding the factors responsible for reduced utilization of revascularization in female patients with STEMI.

We hypothesized that non‐Caucasian women from ethnic and racial minority backgrounds would have worse clinical outcomes with STEMI compared with Caucasian women patients. We aim to evaluate demographics, in‐hospital outcomes, and revascularization rate differences, based on gender and ethnic difference.

## 2 MATERIAL AND METHODS

### 2.1 Database description

Patients were selected from the National Inpatient Sample (NIS) database, which is part of the Healthcare Cost and Utilization Project (HCUP), sponsored by the agency for Healthcare Research and Quality (AHRQ).^10^ It was designed as a stratified probability sample to be representative of all nonfederal acute care hospitals in the United States. Hospitals are stratified according to ownership/control, bed size, urban/rural location, teaching status, and geographical region. A 20% probability sample of all hospitals within each stratum is collected and weighted to ensure that they are nationally representative. In 2016–2018, the NIS included around 4575 hospitals in 47 states, covering around 97% of the US population, making the database the largest in the United States.^11^ The NIS reports data using the ICD‐10‐CM (Medical diagnosis), and PCS (Procedures) codes. The NIS data have been used to provide reliable cardiovascular burden estimates.^4^, ^12^, ^13^ This study was exempt from the Institutional Review Board evaluation, as the data is deidentified and publicly available.

### 2.2 Design and population

This is a retrospective cohort study of female adult patients hospitalized from January 1, 2016, to December 31, 2018, with a primary admission diagnosis of STEMI. Patients were excluded if they were younger than 18 years of age or were admitted with another primary diagnosis. We further divided our population by race into Caucasian, African American, Hispanic, Asian, and other races (Native Americans, and other races not specified). Racial minorities is a collective term used in this paper and is defined as all non‐Caucasian races (including African American, Hispanics, Asian, and other minority races).

### 2.3 Variables

Mortality, length of stay (LOS), total hospitalization charges/costs, and disposition were extracted from the NIS for each hospitalization. Complications were identified using the proper ICD‐10‐CM and PCS codes. Potential confounders included in our multivariate logistic regression analysis were gender, age, race, median yearly income, patients zip code, Charlson comorbidity index, hospital location (rural or urban), geographic region (Northeast, West, South or Midwest), hospital teaching status, and hospital bed‐size included with each NIS discharge. The severity of comorbid conditions was defined using the modified Charlson's comorbidity index (CCI), which contains 17 weighted comorbid conditions with a score ranging from 0 to 33.^14^, ^15^ A higher score is related to a higher burden of comorbid conditions before the index admission. Appropriate weights were provided by the AHRQ/HCUP to generate national estimates.

### 2.4 Outcomes

The primary outcome was in‐hospital mortality during the index admission. Secondary outcomes were healthcare utilization (LOS, total hospitalization charges, and cost), and post‐procedural complications including, mechanical ventilation for less than 24 h, mechanical ventilation for 24–96 h, mechanical ventilation for more than 96 h, Acute Kidney Injury (AKI) requiring renal replacement therapy (RRT), post‐procedural cerebrovascular accident (CVA), cardiac arrest, postprocedural related bleeding, use of vasopressors, acute liver failure, acute right ventricular failure, skilled nursing facility (SNF) or home healthcare (HHC) transfer, and post‐procedural blood transfusion. We also evaluated the use of PCI, fibrinolytic, and CABG, pulmonary artery catheter (PAC), intra‐aortic balloon pump (IABP), and impella use among races.

### 2.5 Statistical analysis

Analyses were performed using STATA version 16 (College Station, Texas). The NIS is based on a complex sampling design that includes weighting, clustering, and stratification. STATA facilitates analysis to produce nationally representative unbiased results, variance estimates, and *p*‐values. We included an extensive multivariate logistic regression analysis to adjust for covariate imbalance, selection bias, and potential confounders which we deem non‐inferior to a propensity score with covariate adjustment based on prior studies comparing both methods.^16^ Proportions were compared by using the Fischer exact test, and continuous variables were compared by using the student *t*‐test. All *p*‐values were two‐sided with .05 as a threshold for statistical significance. The study adheres to best methodological practices for the NIS analysis to minimize the NIS limitations and to provide reliable results.^17^, ^18^

## 3 RESULTS

### 3.1 Patient characteristics

**Figure 1:**
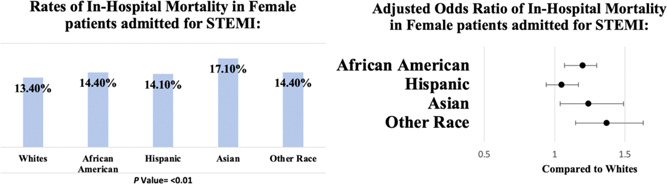
Comparison of in‐hospital mortality in women for different races admitted with STEMI: Rates of in‐hospital mortality in female patient admitted for STEMI according to races. Adjusted odds ratio for in‐hospital mortality of women patients admitted for STEMI. AKI, acute kidney injury; CABG, coronary artery bypass graft; HHC, home health care; IABP,  intra‐aortic balloon pump; LOS, length of stay; MCS, mechanical circulatory support; PCI, percutaneous coronary intervention; RRT, renal replacement therapy; SNF, skilled nursing facility; STEMI, ST‐elevation myocardial infarction.

We identified 642 665 with STEMI admissions, of whom 208,223 were females (32.6%). Of those women, 75.7% were Caucasian, 11.3% African American, 7.4% Hispanic, 2.4% Asian, and 4.3% other races. African American and Hispanic patients were comparatively younger than Caucasian patients. African Americans, Hispanics, and Asians had higher comorbidity scores when compared with Caucasians. African Americans and Hispanics had lower median annual income when compared with Caucasian patients. Non‐Caucasians had lower utilization of Medicare as primary payer and higher use of Medicaid when compared with Caucasians. Caucasian females had a lower incidence of type 2 diabetes mellitus (DM2), chronic kidney disease (CKD), end‐stage renal disease (ESRD), and obesity when compared with other races. Atrial fibrillation, hyperlipidemia, chronic obstructive pulmonary disease (COPD), peripheral vascular disease (PVD), hypothyroidism, and carotid artery disease, was more prevalent in Caucasians when compared to other races. (Tables 1, 2, and Figure 1).

**Table 1:** Patient and hospital characteristics: STEMI Admission in females from 2016 to 2018.

Race	White	African American	Hispanic	Asian	Another race	*p*‐Value
Number of patients 208 223 (%)	75.7%	11.3%	7.4%	2.4	4.3	N/A
Mean age	69.3	63.7	66.5	70.4	65.3	<.01
Weekend admission	26.7	29.1	25.6	27.8	26.1	<.01
Charlson Comorbidity Index score (%)						
1	24.1	17.2	18.0	17.3	22.3	<.01
2	29.0	25.3	29.0	28.9	30.5	<.01
≥3	47.1	57.5	55.0	54.0	47.2	<.01
Median annual income in patient's zip code, US$ (%)						
1–42 999	27.0	55.3	38.1	13.4	32.6	<.01
43 000–53 999	29.0	22.0	25.1	18.5	22.7	<.01
54 000–70 999	24.4	14.5	21.4	27.2	21.2	<.01
>71 000	18.3	14.5	21.4	27.2	21.2	<.01
Insurance type, (%)						
Medicare	64.0	53.1	51.2	55.6	50.2	<.01
Medicaid	8.0	17.6	19.6	17.6	15.4	<.01
Private	22.3	20.5	19.7	19.5	21.41	<.01
Self‐pay	3.6	5.9	7.2	4.4	7.2	<.01
Hospital location (%)						
Northeast	19.0	15.0	14.0	17.9	20.1	<.01
Midwest	26.0	20.9	5.6	7.1	14.5	<.01
South	39.2	54.7	43.4	17.5	39.0	<.01
West	16.0	9.4	36.9	57.5	26.3	<.01
Hospital bed size, and characteristics (%)						
Small	17.0	15.5	16.3	13.0	15.7	.10
Medium	29.5	29.9	33.2	33.1	27.4	.02
Large	53.5	54.7	50.5	53.9	56.9	.05
Urban hospital	90.5	95.2	98.1	98.0	92.0	<.01
Teaching hospital	65.7	78.5	72.1	75.0	69.2	<.01
Healthcare utilization resources						
Length of stay, mean; days	4.7	5.9	5.3	5.5	5.2	<.01
Total charges, mean; US$	100 532	111 438	139 806	142 898	124 442	<.01
Total costs, mean; US$	23 847	25 796	27 398	32 059	29 390	<.01

Abbreviation: STEMI, ST‐elevation myocardial infarction.

**Table 2:** Patient comorbidities, and inpatient outcomes: STEMI Admission in females from 2016 to 2018.

Race	White	African American	Hispanic	Asian	Another race	*p*‐Value
Comorbidities (%)						
T2DM	30.5	41.2	52.3	47.8	40.5	<.01
Cirrhosis	0.4	0.5	1.3	0.5	0.5	<.01
Dyslipidemia	59.1	55.1	58.9	58.5	57.6	<.01
Atrial fibrillation	20.0	12.6	15.6	19.6	14.6	<.01
COPD	18.3	13.0	9.7	7.4	11.9	<.01
HTN	47.2	47.8	47.2	45.0	48.2	.56
PVD	5.2	4.8	3.6	2.5	3.8	<.01
Hypothyroid	18.6	7.7	14.0	11.9	13.0	<.01
CKD	15.5	24.0	19.6	21.7	16.9	<.01
ESRD	1.6	7.5	5.5	6.7	4.0	<.01
Carotid artery disease	1.6	1.0	1.0	1.1	1.3	<.01
CAD	70.6	67.2	70.6	70.3	71.3	<.01
OSA	5.2	6.0	3.9	2.2	4.3	<.01
Prior MI	10.0	10.9	10.2	9.0	11.0	.12
Prior PCI	12.0	11.4	11.8	9.3	11.8	.29
Prior CABG	4.2	4.0	4.7	3.6	4.0	.51
Prior CVA	7.2	8.5	7.0	7.6	6.0	<.01
Obesity	17.1	23.0	18.8	8.6	16.5	<.01
Tobacco abuse	1.7	2.1	1.3	0.8	2.4	<.01
Inpatient outcomes (%)						
Respiratory failure/mechanical ventilation	13.2	18.5	16.8	19.4	17.3	<.01
Mechanical ventilation <24 h	5.3	6.0	5.9	6.8	6.5	.01
Mechanical ventilation 24 h–96 h	5.0	6.7	6.5	7.7	6.3	<.01
Mechanical ventilation >96 h	3.2	6.1	4.9	5.1	4.8	<.01
PCI	57.4	51.2	53.7	50.5	57.7	<.01
Fibrinolytics	1.1	0.94	1.3	1.1	1.3	.65
CABG	3.6	3.0	4.2	4.4	4.8	<.01
AKI with RRT	0.9	1.7	2.3	1.2	1.5	<.01
Blood transfusion	5.0	7.5	7.0	10.4	5.9	<.01
Procedural related bleeding	1.8	1.3	1.7	2.3	1.8	.23
Acute right ventricular failure	0.1	0.1	0.3	0.2	0.2	.05
Acute hepatic failure	3.1	3.7	4.0	4.1	4.4	<.01
Vasopressors	2.7	3.2	3.1	4.0	3.4	.04
Cardiogenic shock	12.6	11.5	13.6	15.1	16.0	<.01
Use of IABP	5.9	5.3	6.8	6.8	8.7	<.01
Use of Impella	1.4	1.7	1.6	1.8	2.2	.03
LVAD	1.2	1.5	1.5	1.2	2.3	<.01
PAC	0.6	0.5	0.7	0.2	0.5	.41
Cardiac arrest	0.2	0.2	0.3	0.4	0.4	.19
SNF transfer	20.1	19.1	16.7	19.4	17.3	<.01
HHC transfer	11.2	11.4	12.8	15.4	10.1	<.01
Died during hospitalization	13.4	14.4	14.1	17.1	14.4	<.01

Abbreviations: AKI, acute kidney injury; CABG, coronary artery bypass graft; CAD, coronary artery disease; CKD, chronic kidney disease; COPD, chronic obstructive pulmonary disease; CVA, cerebrovascular accident; ESRD, end‐stage renal disease; HHC, home health care; HTN, hypertension; IABP, intra‐aortic balloon pump; LVAD, left ventricle assisted device; MI, myocardial infarction; OSA, obstructive sleep apnea; PAC, pulmonary artery catheter; PCI, percutaneous coronary intervention; PVD, peripheral vascular disease; RRT, renal replacement therapy; SNF, skilled nursing facility; STEMI, ST‐elevation myocardial infarction; T2DM, type 2 diabetes mellitus.

### 3.2 Median annual income

55.3% of African Americans had a median annual income <$42 999. This was significantly higher than Caucasians (27%). In addition, only 14.5% of the African Americans had a median annual income greater than $71,000 when compared to Caucasians (18.3%).

### 3.3 Inhospital mortality

Caucasian women had a mortality rate of 13.4%, compared with African Americans (14.4%), Hispanics (14.1%), Asians (17.1%), and other races (14.4%). (Table 2). In our multivariate regression analysis, female patients from African American (aOR 1.2, CI: 1.07–1.30; *p* < .01), Asian (aOR 1.24, CI: 1.04–1.49; *p* < .01), and other races (aOR 1.37, CI: 1.15–1.63; *p* < .01) had higher odds of in hospital mortality when compared with Caucasian females. There was no difference in mortality between Caucasian and Hispanic women (Tables 2 and 3; Figure 1).

**Table 3:** Adjusted odds ratio for primary and secondary outcomes.

	Adjusted odds ratio	95% CI	*p*‐Value
Mortality			
*Whites (Reference)*			
African Americans	1.2	1.07–1.30	<.01
Hispanics	1.05	0.94–1.17	.36
Asian	1.24	1.04–1.49	.01
Another race	1.37	1.15–1.63	<.01
LOS			
Whites (Reference)			
African Americans	+0.69 days	0.45–0.94	<.01
Hispanics	+0.31 days +0.59 days	0.33–0.59	.02
Asian	+0.49 days	0.12–1.07	.01
Another race		0.10–0.80	.01
Total charges ($)			
Whites (Reference)			
African Americans	+633 $	−4194 to +5461	.79
Hispanics	+20 840	+13 462 to 28 217	<.01
Asian	+18 633	+6964 to +30 301	<.01
Another race	+14 720	+5989 to +23 543	<.01
Total costs ($)			
Whites (Reference)			
African Americans	+122	−803 to +1047	.79
Hispanics	+974	−382 to +2331	.15
Asian	+4152	+1598 to +6705	<.01
Another race	+3874	+1897 to 5861	<.01
Respiratory failure/mechanical ventilation			
Whites (Reference)			
African Americans	1.12	0.98–1.29	.09
Hispanics	1.05	0.88–1.25	.57
Asian	1.19	0.91–1.55	.18
Another race	1.29	1.05–1.59	.01
Mechanical ventilation >24 h			
Whites (Reference)			
African Americans	1.30	1.17–1.45	<.01
Hispanics	1.22	1.06–1.39	<.01
Asian	1.48	1.21–1.181	<.01
Another race	1.35	1.14–1.60	<.01
AKI with RRT			
Whites (Reference)			
African Americans	1.41	1.08–1.83	.01
Hispanics	1.88	1.43–2.48	<.01
Asian	1.06	0.58–1.90	.85
Another race	1.45	0.94–2.24	.08
Post‐PCI related bleeding			
Whites (Reference)			
African Americans	0.72	0.62–0.85	<.01
Hispanics	0.94	0.80–1.11	.52
Asian	1.12	0.86–1.46	.37
Another race	0.94	0.76–1.16	.59
Blood transfusion			
Whites (Reference)			
African Americans	1.40	1.22–1.59	<.01
Hispanics	1.34	1.14–1.59	<.01
Asian	1.94	1.54–2.45	<.01
Another race	1.42	1.15–1.73	<.01
Cardiac arrest			
Whites (Reference)			
African Americans	0.97	0.45–20.6	.93
Hispanics	1.58	0.76–3.29	.21
Asian	2.07	0.73–5.8	.17
Another race	0.31	0.44–2.29	.25
SNF/HHC transfer			
Whites (Reference)			
African Americans	1.17	1.08–1.26	<.01
Hispanics	1.02	0.92–1.12	.64
Asian	1.10	0.94–1.28	.19
Another race	1.01	0.90–1.14	.75
MCS (Impella, and/or IABP)			
Whites (Reference)			
African Americans	0.84	0.74–0.96	.01
Hispanics	1.10	0.95–1.27	.19
Asian	1.16	0.90–1.48	.23
Another race	1.53	1.30–1.79	<.01
Cardiogenic shock			
Whites (Reference)			
African Americans	0.79	0.73–0.87	<.01
Hispanics	0.95	0.87–1.05	.40
Asian	1.04	0.89–1.22	.56
Another race	1.12	0.99–1.25	.05
PCI			
Whites (Reference)			
African Americans	0.67	0.62–0.72	<.01
Hispanics	0.81	0.74–0.88	<.01
Asian	0.79	0.69–0.90	<.01
Another race	0.90	0.81–1.01	.07
CABG			
Whites (Reference)			
African Americans	0.63	0.51–0.76	<.01
Hispanics	1.01	0.83–1.24	.87
Asian	1.18	0.85–1.65	.31
Another race	1.25	0.98–1.59	.06

Abbreviations: AKI, acute kidney injury; CABG, coronary artery bypass graft; HHC, home health care; IABP, intra‐aortic balloon pump; LOS, length of stay; MCS, mechanical circulatory support; PCI, percutaneous coronary intervention; RRT, renal replacement therapy; SNF, skilled nursing facility; STEMI, ST‐elevation myocardial infarction; T2DM, type 2 diabetes mellitus.

### 3.4 Length of stay

The mean length of stay was 4.7 days for Caucasian patients, 5.9 days for African Americans, 5.3 days for Hispanics, 5.5 days for Asians, and 5.2 days for patients from other races. In our multivariate regression analysis non‐Caucasians had higher odds for a longer length of stay when compared to Caucasians; for example, African Americans (+0.69 days, CI: 0.45–0.94 days; *p* < .01), Hispanic (+0.3 days, CI: 0.33–0.58 days; *p* = .02), Asians (+0.59 days, CI: 0.12– 1.07 days; *p* < .01), and other races (+0.49 days; CI: Extra 0.10–0.80 days; *p* < .01) (Tables 2 and 3).

### 3.5 Total hospitalization cost and charges

AHRQ‐ HCUP defines hospital charges as the amount that the hospital bills for an admission and the hospital costs as the total expense incurred in the production of all hospital services.^19^ Caucasian women had lower total hospitalization charges and cost when compared with all other races. The mean hospitalization charges were $100, 532 for Caucasian females, $111 438 for African Americans, $139 806 for Hispanics, $142 898 for Asians, and $124 442 for females of other races. The mean hospitalization cost was $23 847 for Caucasians, $25 796 for African Americans, $27 398 for Hispanics, $32 059 for Asian and $29 390 for other races. After our multivariate regression analysis Hispanics, Asian, and other races had significantly higher total cost when compared with Caucasians (Tables 2 and 3).

### 3.6 Use of PCI, CABG, fibrinolytics, and mechanical support devices

**Figure 2:**
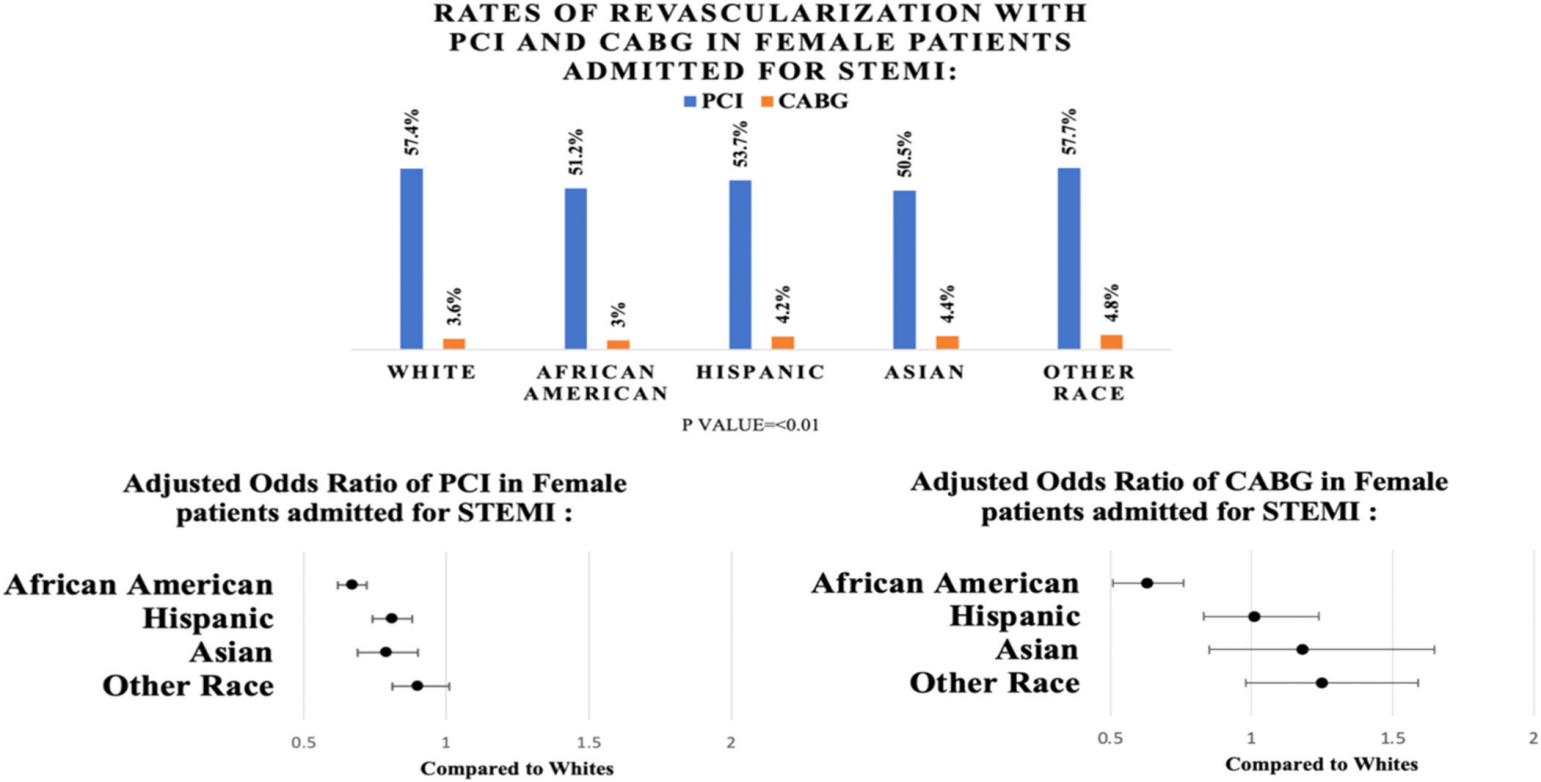
Comparison of rates of revascularization among women of different races admitted with STEMI in 2016–2018: Rates of revascularization with PCI or CABG in female women admitted for STEMI according to races. Adjusted odds ratio for PCI and CABG in women patients admitted for STEMI according to races. CABG, coronary artery bypass graft; PCI, percutaneous coronary intervention; STEMI, ST‐elevation myocardial infarction.

White patients were more likely to have PCI during the index admission. PCI rates were 57.4% for Caucasians, 51.2% for African Americans, 53.7% for Hispanics, 50.5% for Asians, and 57.7% for other races. In our multivariate regression analysis Caucasian patients has significantly higher PCI rate compare with non‐Caucasian. The rates of CABG were 3.6% for Caucasians, 3% for African Americans, 4.2% for Hispanic, 4.4% for Asians, and 4.8% for other races. When compared to Whites, African Americans had less odds of receiving CABG, and Mechanical circulatory support during index admission (Tables 2 and 3; Figure 2).

### 3.7 Inhospital complications

Caucasian patients had a lower rate of acute respiratory failure, mechanical ventilation, long‐term mechanical ventilation, acute kidney injury requiring renal replacement therapy, acute hepatic failure, and use of vasopressors when compared with other races. In our multivariate regression analysis African Americans, Hispanics, Asians, and other races had higher odds of long‐term mechanical ventilation (>24 h), AKI requiring RRT, post‐procedural blood transfusion, when compared with Caucasians. Cardiogenic shock (CS) was more prevalent in Hispanic, Asians, and other races when compared with Caucasians, and African Americans, and after adjusting for confounders African Americans had less odds of cardiogenic shock when compared with Caucasians. There was no statistically significant difference in other complications when compared with Caucasians (Tables 2 and 3).

## 4 DISCUSSION

In the present study, we demonstrate the effect of racial difference in clinical and procedural outcomes in women patients admitted for STEMI. We found that women from minority groups have higher odds of in‐hospital mortality and lower odds of percutaneous coronary intervention when compared with Caucasian women. So far, there have been reports studying the difference in outcomes in STEMI patients between the different genders and between the different races. Our study is the only one so far that we have found that is focusing on the differences in women presenting with STEMI among the different races. Minorities represent a 25% of patients admitted with STEMI (11.3% African American, 7.4% Hispanics, 2.4% Asian, and 4.3% another race). Non‐Caucasians were more likely to be younger compared with Caucasian group. NonCaucasian group also had higher Charlson comorbidity index, higher use of urban and teaching hospitals, with longer LOS, total charges, total cost, higher incidence of type 2 diabetes mellitus, ESRD, CKD and Obesity. Non‐Caucasian groups, however, had a lower yearly income, lower use of Medicare compared with Caucasians. There are data that support these finding that 15%–20% of women with STEMI are minorities and tend to have more comorbidities that increase risk of MACE after a STEMI.^4^, ^20^, ^21^

Non‐Caucasian females in our study developed more in‐hospital complications when compared with Caucasian females and after adjustment non‐Caucasians have higher odds of acute respiratory failure requiring mechanical ventilation, long term mechanical support, AKI requiring RRT, higher use of vasopressors and need for blood transfusion. Data from Chet et al.,^22^ and other observational studies have demonstrated that minorities and especially African Americans tend to have more bleeding complications, contrast induce nephropathy, and other complications post PCI.^23^ Data from retrospective studies have demonstrated that this could be secondary to late presentation or lack of early intervention as compared with Caucasians.^8^, ^24^, ^25^, ^26^ In the National healthcare and disparities report there is documented a large racial and ethnic disparities that lead to delay and access to medical care among minorities when compared with Caucasians.^27^

Non‐Caucasians were initially found to have higher rate of cardiogenic shock at presentation compared with Caucasians. After adjusting for baseline characteristics and clinical features, this was not found to be statistically significant. In fact, after adjusting, the incidence of cardiogenic shock post STEMI was found to be lower in African American women when compared to white women. The use of mechanical circulatory support was also as expected found to be lower in African Americans (*p* .01). This was higher in those all included in “other races.” Ya'qoub et al.,^4^ noted that female patients were less likely to undergo MCS as well as right heart catheterizations when compared with Caucasian men. Our study contrastingly honed in on the differences between women of different races and may in part explain the reason for the different findings. This lower incidence of cardiogenic shock is not completely understood but there is data that the use of MCS in African American patients is lower than Caucasians and other races.^4^, ^28^ Previous studies have also noted lower utilization of right heart catheterization in women.^28^ As a whole, underdiagnoses of cardiogenic shock in addition to inadequate use of right heart catheterizations to optimally manage cardiogenic shock with MCS may explain our findings.

In terms of healthcare utilization non‐Caucasians had higher length of stay with almost an extra day when compared with Caucasians, a difference that was significant after adjusting for confounders. Total hospital charges and cost were higher in non‐Caucasians and persisted after adjusting for confounders in charges in Hispanics, Asian and other races when compared with Caucasian. Asian and other races had significant higher total hospitalization cost when compared with Caucasians. This was demonstrated in prior studies using the National Inpatient Sample and NCDR where minorities had higher length of stay and hospitalization charges for admission for acute coronary syndromes when compared with Caucasians that could be explained by higher complications and comorbidities.^22^, ^23^, ^29^, ^30^

Revascularization rates with PCI during STEMI were lower in non‐Caucasians when compared with Caucasians. After adjustment for confounders African American (aOR 0.67; *p* < .01), Hispanics (aOR 0.81; *p* < .01), and Asians (aOR 0.79; *p* < .01) had lower odds of revascularization with PCI when compared with Caucasian patients after a STEMI presentation. This important finding demonstrates an important gap in care for minority groups that has already been reported before where minorities groups have less access to prompt PCI when compared with Caucasian population regardless of insurance status.^31^, ^32^, ^33^, ^34^, ^35^

African Americans had an overall lower odds of undergoing CABG (aOR 0.63; *p* < .01) for STEMI when compared with Caucasians. No statistically significant differences were found in the rates of use of CABG among other races. Similar findings have been reported in prior studies where African Americans had lower odds of CABG for chronic and acute coronary syndromes when compared with Caucasians.^20^, ^36^ Previous studies have thought this difference was attributed partly to lower surgical referrals and partly due to a higher mortality attributed to more complex anatomy, higher incidence of graft failure and lesser likelihood of successful revascularization in women.^36^ Our study has used a deidentified database with limited to no information regarding the complexities entailing individual management and further studies are required to identify the reasons for the findings noted.

Non‐Caucasians had higher in‐hospital mortality when compared with Caucasians (14.4% for African Americans, 14.1% for Hispanics, 17.1% for Asians, 14.4% for other races and 13.4% for Whites). After adjusting for confounders, African Americans (aOR 1.2; *p* < .01), Asians (aOR 1.24; *p* < .01), and other races (aOR 1.37; *p* < .01) had higher odds of mortality when compared with Caucasians. This had been demonstrated in prior studies from MEDICARE and the NIS databases where African Americans had higher in‐hospital mortality when compared with Caucasians^4^, ^36^, ^37^ and similar difference in Asian patients in data from the AHRQ HCUP database.^38^ Data from the CRUSADE registry and NCDR ACTION registry have demonstrated no difference in mortality after ACS between Hispanics and Whites.^39^, ^40^ This mortality difference could be explained by the fact that minorities have higher comorbidities, complications and lack of early access to medical treatment, and by our findings that minorities have lower odds of revascularization, and in African Americans lower odds of MCS utilization.

We did note that a higher percentage of African American women belong to families with lower annual median income. Educational information of these women could not be found. Further studies are needed to identify if the socioeconomic background of women of different races impact their clinical outcome.

Our findings demonstrate significant disparities in the care of non‐Caucasian females after STEMI admission with lower rates of PCI, CABG, and mechanical support devices, higher healthcare utilization and mortality when compared to Caucasian women. While we already know factors that could be responsible for these poor outcomes, including higher incidence of risk factors for CAD, economic disparity, lack of access to specialized facility and poor follows up. Further studies are needed to understand the cause‐ effect relationship for these differences in treatment approach to reduce barriers to optimal care and improved outcomes. In addition, a higher representation of minority groups in randomized clinical trials through aggressive enrolling will assist with defining protocols tailored specifically towards optimal outcomes in these population groups.

## 5 LIMITATIONS

The inherent limitations for this study are its retrospective nature and the use of an administrative database. The NIS analysis that is based on ICD‐10 codes, could be prone to coding errors, underreporting comorbidities, using wrong diagnosis/procedure codes. However, this database has been internally validated extensively to report clinical and procedure characteristics. Another limitation is that the NIS lacks long‐term outcomes. Due to the complexity of the database, we do not have information on the STEMI severity, hemodynamic variables, or procedure related details. In addition, studying the impact of socioeconomic factors and lifestyle differences among the differences races on the outcome of these women presenting with STEMI is out of the scope of this article. Being an observational retrospective study, there remains a potential for selection bias and unmeasured confounders. In this study, we adhere to required practices for the NIS and performed an extensive multivariate regression analysis to mitigate these risks and deliver reliable results. Despite the database limitations, our study addresses an important knowledge gap to understand the differences in the outcomes, for female patients admitted for STEMI, based on racial and ethnic difference.

## 6 CONCLUSION

Our analysis demonstrates that non‐Caucasian women represent a 25% of female patients presenting for STEMI. Non‐Caucasian groups were found to have a lower rate of and higher mortality compared with Caucasian women. These inequalities should be further investigated to design protocols to optimize care and close the gap in cardiovascular outcomes.

## CONFLICT OF INTEREST STATEMENT

The authors declare that there is no conflict of interest.

## Data Availability

Data sharing is not applicable to this article as no new data were created or analyzed in this study. Data available within the article or its supplementary materials.
